# A high-throughput-sequence analysis infrastructure technology investigation framework for the evaluation of next-generation sequencing software

**DOI:** 10.1186/gb-2011-12-s1-p48

**Published:** 2011-09-19

**Authors:** Xiaoyu Liu, Sumit Middha, Steven Hart, Asha Nair, Ahmed Hadad, Patrick Duffy, Jean-Pierre Kocher

**Affiliations:** 1 Division of Biomedical Statistics and Informatics, Mayo Clinic, Rochester, MN 55905, USA

## 

High-throughput sequencing (HTS) is an emerging technology that promises to deliver unparalleled information on genomic variations. As technology evolves and matures, and as a deeper understanding of this technology is gained, new and upgraded tools for analyzing HTS will become available and will need to be evaluated and validated. To facilitate this cumbersome task, we have developed an HTS validation framework into which both in-house-generated synthetic datasets and well-characterized experimental datasets have been incorporated for controlled testing and evaluation of these analysis tools. Currently, the framework can be used to assess algorithms for short-read mapping, variant calling and RNA-Seq-derived gene expression measurements. The framework is deployed in the Amazon EC2 cloud so that it is available to the broader research community. Using our framework, researchers can further validate interfaced applications with preferred parameters, upload their own datasets for processing, and interface new applications with the framework for validation and comparison.

We report the performance of several alignment, variant calling and RNA-Seq analytic tools that have been tested with our framework. We also provide feedback on the challenges and benefits of Amazon EC2 deployment.

